# Effects of an SGLT Inhibitor on the Production, Toxicity, and Elimination of Gut-Derived Uremic Toxins: A Call for Additional Evidence

**DOI:** 10.3390/toxins14030210

**Published:** 2022-03-15

**Authors:** Pieter Evenepoel, Bjorn Meijers, Rosalinde Masereeuw, Jerome Lowenstein

**Affiliations:** 1 Laboratory of Nephrology, KU Leuven Department of Microbiology and Immunology, University Hospitals Leuven, 3000 Leuven, Belgium; bjorn.meijers@uzleuven.be; 2 European Uremic Toxin Work Group-EUTox, Danube University Krems, 3500 Krems, Austria; r.masereeuw@uu.nl; 3 Division of Pharmacology, Utrecht Institute for Pharmaceutical Sciences, Utrecht University, 3584 CG Utrecht, The Netherlands; 4 Nephrology Division, NYU Langone Medical Center, New York, NY 10016, USA; jerome.lowenstein@nyumc.org

**Keywords:** sodium–glucose cotransporter inhibitors, gut microbial metabolism, cardiovascular, cardiometabolic health

## Abstract

Sodium–glucose cotransporter (SGLT) inhibitors are a class of oral hypoglycemic agents, which, in recent years, have been shown to improve renal and cardiovascular outcomes in patients with diabetic and non-diabetic chronic kidney disease. There remains considerable debate regarding the potential glucose-independent mechanisms by which these benefits are conferred. SGLT inhibitors, to a variable extent, impair small intestinal glucose absorption, facilitating the delivery of glucose into the colon. This suppresses protein fermentation, and thus the generation of uremic toxins such as phenols and indoles. It is acknowledged that such a shift in gut microbial metabolism yields health benefits for the host. SGLT inhibition, in addition, may be hypothesized to foster the renal clearance of protein-bound uremic toxins. Altered generation and elimination of uremic toxins may be in the causal pathway between SGLT inhibition and improved cardiometabolic health. Present review calls for additional research.

## 1. SGLT Inhibitors

Sodium–glucose cotransporter (SGLT; cotransporter from the *SLC5* family) inhibitors are a potent class of oral hypoglycemic agents that have been shown to improve renal and cardiovascular outcomes in patients with diabetic and non-diabetic chronic kidney disease. In humans, the family of SGLT comprises at least six different isoforms. Of these, SGLT1 (*SLC5A1*) and SGLT2 (*SLC5A2*) have been studied extensively because of their fundamental role in the transepithelial transport of glucose and sodium in the small intestine and the kidney, through an active mechanism exploiting the Na^+^–electrochemical gradient generated by active sodium extrusion by the basolateral Na^+^/K^+^-ATPase. Under baseline conditions, SGLT1 is responsible for glucose absorption in the small intestine, and for the reabsorption of nearly 10% of the filtered glucose load in the renal proximal tubule segment 3. SGLT2, conversely, is primarily expressed at the apical membrane of the renal proximal tubule segments 1 and 2 and is responsible for the reabsorption of approximately 90% of the filtered glucose load [1]. In the past decade, several SGLT inhibitors became commercially available or are in the final phase of development [2]. Interest has been focused on inhibitors specifically targeting SGLT2. These inhibitors are referred to as SGLT2 inhibitors and encompass canagliflozin, dapagliflozin, empagliflozin, ertugliflozin, ipragliflozin, and tofogliflozin. Importantly, SGLT2 inhibitors show variable cross-reactivity with SGLT1 (Table 1), which, in a clinical setting, seems to be relevant only for canagliflozin [3]. Sotagliflozin acts on both SGLT1 and SGLT2 and is referred to as a dual SGLT inhibitor.

SGLT2 inhibition lowers the renal glucose threshold to approximately 100 mg/dL, thereby promoting urinary glucose excretion without increasing the risk of hypoglycemia. Currently, the available SGLT2 inhibitors share several pharmacokinetic characteristics (Table 1) and show comparable effects on glycemic control. The drugs act from the luminal side of the tubular cells. They can reach this side by glomerular filtration and/or tubular secretion, probably through organic anion transporters [11,14]. The latter could facilitate high local concentrations in the unstirred layer of the apical brush border membrane. SGLT2 inhibitors are well absorbed and are modestly–strongly bound to plasma proteins (Table 1). Except for empagliflozin and tofogliflozin, the renal clearance of most SGLTs is low and only a small proportion of the dose is recovered in urine as parent compound. Glomerular filtration of the free fraction—and tubular secretion to a variable extent—contribute to luminal concentrations, and as such determine the therapeutic response. 

**Figure 1:**
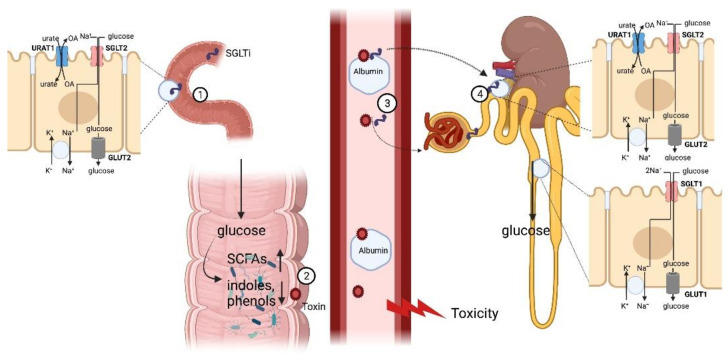
Interaction of SGLT with gut-derived, protein-bound uremic toxins. (1) SGLT inhibitors to a variable extent impair glucose absorption in the small intestine. (2) Glucose entering the large intestine will cause a shift from proteolytic toward saccharolytic fermentation, decreasing the exposure of the host to potentially toxic phenols and indoles. (3) Within the circulation gut-derived protein-bound uremic toxins may alter binding of SGLT inhibitors to albumin. (4) In the kidney, SGLT inhibitors may affect renal handling of protein-bound uremic toxins and vice versa and affect renal urate excretion. Abbreviations: see body of manuscript. Created with BioRender.com (accessed on 4 January 2022).

SGLT inhibitors inhibit SGLT1 also preferentially from the extracellular side. Because of more extensive protein binding (Table 1), the clinical dosage of canagliflozin is higher (100–300 mg) than for other SGLT2 inhibitors [15]. Consequently, the luminal concentration of canagliflozin in the upper small intestine after oral administration may hypothesized to transiently exceed the K_i_ value for SGLT1 (i.e., approximately 17 nM [16]). This implies that canagliflozin could inhibit SGLT1 from the luminal side of the intestine. SGLT1 inhibition appears to delay and impair glucose absorption and might also influence water transport [17]. Consequences include reduced postprandial glucose levels, enhanced glucose-induced plasma glucagon-like peptide 1 (GLP1) secretion, and increased colonic delivery of glucose. The latter may result in a more favorable microenvironment, promoting the production of short-chain fatty acids (SCFAs), while suppressing protein fermentation (Figure 1) [18,19]. 

## 2. The History of the Discovery and Description SGLT1 and SGLT2

Phlorizin is a glycoside that can be found in the bark and roots of several fruit trees, including apple and pear. In 1885, it was discovered by von Mering that phlorizin induces transient glucosuria. It is poorly adsorbed from the gastrointestinal tract, and intravenous administration is needed to exert its physiological effects. More recently, it has been shown that phlorizin functions as an SGLT inhibitor [20]. Our understanding of the role of SGLT-inhibitors dates back to an era before molecular biology. In his magnum opus, *The kidney: structure and function in health and disease* (Oxford University Press, Oxford, UK, 1951), Homer W. Smith provided a description of the physiological effects of phlorizin, noting that it partly blocks the reabsorption of vitamin C and, of note, it depresses the tubular excretion of phenol red in chicken. It was speculated that this effect was the consequence of diversion of energy rather than a specific interference with the transporter mechanisms. These early speculations have not been corroborated by any recent data grounded in molecular biology.

## 3. SGLT Inhibitors and Renal and Cardiovascular Outcomes

In the last decade, four SGLT2 inhibitors have become been granted marketing authorization by the European Medicines Agency and the US Food and Drug Administration for management of hyperglycemia in type 2 diabetes. An increasing number of clinical trials have reported beneficial cardiovascular and renal outcomes among diabetic and non-diabetic patients receiving SGLT2 inhibitors [21,22,22,23,24] or sotagliflozin [25]. The benefits with respect to heart failure and chronic kidney disease (CKD) have been consistent across all commercially available SGLT inhibitors and, importantly, were independent of glucose lowering. The glucose-independent mechanisms by which these benefits are conferred remain a matter of ongoing debate. Hemodynamic effects, changes in cardiac substrate utilization and mitochondrial function, alterations in the kidney–heart interaction, and increased osmotic diuresis are only few of the potential mechanisms. Further elucidation of the mechanisms driving the cardiorenal benefits of SGLT2 inhibitors is mandatory. Such studies might not be necessary to drive implementation, but they are useful for advancing knowledge that will allow innovation in future therapies. A better understanding of the mechanisms of action will also facilitate communication regarding the rationale for use of these drugs with clinicians and patients [26].

## 4. The Gut–Cardio–Renal Axis

The human intestine hosts a complex and diverse system of mutualistic microorganisms. This rich ecosystem is increasingly regarded as playing a crucial role in human health and disease. Gut microbiota can interact with the host by the production of a diverse array of metabolites [27]. Mounting evidence indicates that gut dysbiosis may link dietary patterns to cardiometabolic diseases. A Western-style diet, characterized by high fat, high animal protein, and low dietary fiber intake may cause microbial metabolism to shift from saccharolytic towards proteolytic fermentation. Such a shift results in less (local and systemic) exposure to SCFAs and higher exposure to indoles and phenols. A Mediterranean diet, conversely, may push microbial metabolism in the opposite direction [28,29]. SCFAs participate in the maintenance of intestinal mucosal integrity, improve glucose and lipid metabolism, increase the secretion of glucagon-like peptide-1 (GLP-1) and peptide YY (PYY), and regulate the immune system and inflammatory responses, as such, they confer protection against cardiometabolic and kidney disease [30]. Conversely, phenols and indoles released in the systemic circulation portend cardiometabolic and renal risks, as demonstrated in many experimental and clinical studies [19,31,32,33,34,35,36]. The toxic systemic effects of phenols and indoles are mediated at least in part by increased intracellular oxidative stress.

## 5. SGLT Inhibitors and the Generation, Elimination, and Toxicity of Gut-Derived Uremic Toxins

### 5.1. SGLT Inhibitors and Microbial Metabolism

Canagliflozin and sotagliflozin, to a variable extent, impair small intestinal glucose absorption, facilitating the delivery of glucose into the colon, which fosters carbohydrate fermentation. The ensuing increased production of SCFAs may cause a reciprocal decline of protein fermentation. Well-designed in vitro studies showed that an increased availability of carbohydrates diminishes microbial amino acid fermentation through its pH-lowering effect, its action as a microbial energy source, and the process of “catabolite repression” [18,37].

A proof of concept has recently been demonstrated in two experimental studies [38,39]. Treatment with canagliflozin (10 mg/kg po) for 2 weeks significantly reduced the plasma levels of p-cresyl sulfate and indoxyl sulfate in mice with kidney failure (compared with the vehicle group a 75% and 26% reduction, respectively). Additionally, canagliflozin significantly increased cecal short-chain fatty SCFAs. Analysis of the cecal microbiota demonstrated that canagliflozin significantly changed microbiota composition in the kidney failure mice [38]. In another experimental study using the same kidney failure model, SGL5213—a novel and potent intestinal SGLT1 inhibitor—ameliorated kidney function and reduced gut-derived uremic toxins (phenyl sulfate and trimethylamine-N-oxide). SGL5213 also ameliorated renal fibrosis and inflammation [39]. As such, the effect of SGLT inhibitors may mimic that of dietary fiber supplements [40], prebiotics [41,42], or acarbose [18]. 

### 5.2. SGLT Inhibitors and Renal Elimination of Protein-Bound Uremic Toxin 

Most of abovementioned uremic toxins originating from gut microbial aromatic amino acid metabolism are organic anions and circulate, strongly bound to albumin. Renal excretion thus occurs mainly through tubular secretion [43]. The first step in renal tubular secretion of organic anions is mediated by transporters in the basolateral membrane (BLM). Primary active Na^+^/K^+^-ATPase (EC 3.6.3.9), secondary active Na^+^-dicarboxylate cotransporter 3 (NaDC3/*SLC13A3*), and tertiary active organic anion transporters (OAT1/*SLC22A6*, OAT2/*SLC22A7*, and OAT3/*SLC22A8*) all are involved [44]. The expression of OAT1 and OAT3 partially overlap with the expression of SGLT2 in the cortical proximal convoluted tubules: OAT1 and OAT3 are located in the BLM and SGLT2 in the brush border at the apical side. Whether SGLT2 inhibition has indirect effects on the tubular secretion of organic anions remains to be investigated. Reabsorption of glucose in the proximal kidney tubule is a secondary active transport. SGLT2 inhibition may thus safeguard energy, fostering other secondary or tertiary active transporters, such as OATs. Furthermore, SGLT2 inhibitors have been reported to lower the serum uric acid level by increasing its renal excretion [45]. This most likely occurs through an indirect interaction with URAT1 (*SLC22A12*), responsible for 99% reabsorption of uric acid from the ultrafiltrate, and GLUT9 (*SLC2A9*). Canagliflozin was shown to acutely increase the fractional urate excretion in mice, an effect that was absent in *Urat1* knockout mice, despite a similar glucosuric response. The role of *Glut9* was less clear, despite a clear upregulation in gene expression by the SLGT inhibitor [46]. Interference with other transporters, however, remains to be demonstrated.

### 5.3. Protein-Bound Uremic Toxins and the Pharmacokinetics and Pharmacodynamics of SGLT-2 Inhibitors

Protein-bound uremic toxins may be hypothesized to affect the pharmacokinetics and pharmacodynamics of SGLT2 inhibitors by interfering with their protein binding and tubular secretion [47]. In line with this hypothesis, the pharmacodynamic response to SGLT2 inhibitors as assessed by urinary glucose excretion was observed to decline along the severity of kidney dysfunction. Importantly, despite this dampened pharmacodynamic response, the glucose-lowering efficacy and safety of SGLT2 inhibitors are similar in patients with mild CKD and individuals with normal kidney function [4].

### 5.4. SGLT-Inhibitors Attenuate Oxidative Stress

Experimental data suggest that empagliflozin attenuates uremic serum (and indoxyl sulfate)-induced generation of endothelial mitochondrial reactive oxygen species. This leads to the restoration of nitric oxide production and endothelium-mediated enhancement of nitric oxide levels in cardiomyocytes. This effect was shown to be largely independent of sodium–hydrogen exchanger-1 [48].

## 6. Future Prospects

In aggregate, evidence suggests a bidirectional interaction between SGLT inhibitors and protein-bound uremic toxins originating from gut microbial metabolism. There is an urgent need for additional experimental and clinical research. The following research questions, among others, should be addressed: does SGLT inhibition lower the generation of gut-derived uremic toxins? Does SGLT inhibition increase the fractional urinary excretion of urate and protein-bound uremic toxins? A better insight into the mechanisms of the actions of SGLT inhibitors may prove useful in identifying patients that may benefit the most from this new class of drugs. As such, their scope may expand from glucose control in diabetes patients to renal protection in patients with diabetic and non-diabetic nephropathy to cardiometabolic protection in the general population. SGLT inhibitors have the potential to become the long-sought adjuvant therapy to tackle protein-bound uremic toxins—many of which originate from gut microbial metabolism—in patients with advanced-stage CKD.

## Figures and Tables

**Table 1:** Selection of SGLT2 inhibitors and their pharmacokinetic properties in humans.

SGLTi	Human SGLT1 (IC_50_ nM)	Human SGLT2 (IC_50_ nM)	Protein Binding (%)	t_1/2_ (h)	F (%)	f_e_ (%)	Refs.
canagliflozin	684	4.4	98	11–13	65	<1%	[4,5]
dapagliflozin	803	1.6	91	12.2	78	<2%	[4]
empagliflozin	8300	3.1	86	11.4–12.4	60–78	11–29	[4,6,7,8]
ertugliflozin	1960	0.9	94	11–18	100	1.5%	[9,10]
ipragliflozin	2329	8.9	96	10–13	90	<1%	[4,11,12]
tofogliflozin	8444	2.9	83	6.8	97.5	76%	[4,13]

Abbreviations: t_1/2_—terminal half-life in plasma; F—bioavailability; f_e_—fraction of dose that was excreted unchanged in urine; IC50—half maximal inhibitory concentration.

## References

[1] Cefalo C.M.A., Cinti F., Moffa S., Impronta F., Sorice G.P., Mezza T., Pontecorvi A., Giaccari A. (2019). Sotagliflozin, the first dual SGLT inhibitor: Current outlook and perspectives. Cardiovasc. Diabetol..

[2] Rieg T., Vallon V. (2018). Development of SGLT1 and SGLT2 inhibitors. Diabetologia.

[3] Ohgaki R., Wei L., Yamada K., Hara T., Kuriyama C., Okuda S., Ueta K., Shiotani M., Nagamori S., Kanai Y. (2016). Interaction of the Sodium/Glucose Cotransporter (SGLT) 2 inhibitor Canagliflozin with SGLT1 and SGLT2. J. Pharmacol. Exp. Ther..

[4] Scheen A.J. (2015). Pharmacokinetics, Pharmacodynamics and Clinical Use of SGLT2 Inhibitors in Patients with Type 2 Diabetes Mellitus and Chronic Kidney Disease. Clin. Pharm..

[5] Liang Y., Arakawa K., Ueta K., Matsushita Y., Kuriyama C., Martin T., Du F., Liu Y., Xu J., Conway B. (2012). Effect of Canagliflozin on Renal Threshold for Glucose, Glycemia, and Body Weight in Normal and Diabetic Animal Models. PLoS ONE.

[6] Ndefo U.A., Anidiobi N.O., Basheer E., Eaton A.T. (2015). Empagliflozin (Jardiance): A Novel SGLT2 Inhibitor for the Treatment of Type-2 Diabetes. Pharm. Ther..

[7] Heise T., Seman L.J., Macha S., Jones P., Marquart A., Pinnetti S., Woerle H.J., Dugi K. (2013). Safety, Tolerability, Pharmacokinetics, and Pharmacodynamics of Multiple Rising Doses of Empagliflozin in Patients with Type 2 Diabetes Mellitus. Diabetes Ther..

[8] Grempler R., Thomas L., Eckhardt M., Himmelsbach F., Sauer A., Sharp D.E., Bakker R.A., Mark M., Klein T., Eickelmann P. (2011). Empagliflozin, a novel selective sodium glucose cotransporter-2 (SGLT-2) inhibitor: Characterisation and comparison with other SGLT-2 inhibitors. Diabetes, Obes. Metab..

[9] Fediuk D.J., Nucci G., Dawra V.K., Cutler D.L., Amin N.B., Terra S.G., Boyd R.A., Krishna R., Sahasrabudhe V. (2020). Overview of the Clinical Pharmacology of Ertugliflozin, a Novel Sodium-Glucose Cotransporter 2 (SGLT2) Inhibitor. Clin. Pharmacokinet..

[10] Mascitti V., Maurer T.S., Robinson R.P., Bian J., Boustany-Kari C.M., Brandt T., Collman B.M., Kalgutkar A.S., Klenotic M.K., Leininger M.T. (2011). Discovery of a Clinical Candidate from the Structurally Unique Dioxa-bicyclo[3.2.1]octane Class of Sodium-Dependent Glucose Cotransporter 2 Inhibitors. J. Med. Chem..

[11] Choi M.-K., Nam S.J., Ji H.-Y., Park M.J., Choi J.-S., Song I.-S. (2020). Comparative Pharmacokinetics and Pharmacodynamics of a Novel Sodium-Glucose Cotransporter 2 Inhibitor, DWP16001, with Dapagliflozin and Ipragliflozin. Pharmaceutics.

[12] Saito M., Kaibara A., Kadokura T., Toyoshima J., Yoshida S., Kazuta K., Ueyama E. (2019). Pharmacokinetic and pharmacodynamic modelling for renal function dependent urinary glucose excretion effect of ipragliflozin, a selective sodium–glucose cotransporter 2 inhibitor, both in healthy subjects and patients with type 2 diabetes mellitus. Br. J. Clin. Pharmacol..

[13] Ohtake Y., Sato T., Kobayashi T., Nishimoto M., Taka N., Takano K., Yamamoto K., Ohmori M., Yamaguchi M., Takami K. (2012). Discovery of Tofogliflozin, a Novel C-Arylglucoside with an O-Spiroketal Ring System, as a Highly Selective Sodium Glucose Cotransporter 2 (SGLT2) Inhibitor for the Treatment of Type 2 Diabetes. J. Med. Chem..

[14] Fu Y., Breljak D., Onishi A., Batz F., Patel R., Huang W., Song P., Freeman B., Mayoux E., Koepsell H. (2018). Organic anion transporter OAT3 enhances the glucosuric effect of the SGLT2 inhibitor empagliflozin. Am. J. Physiol. Physiol..

[15] Devineni D., Polidori D. (2015). Clinical Pharmacokinetic, Pharmacodynamic, and Drug-Drug Interaction Profile of Canagliflozin, a Sodium-Glucose Co-transporter 2 Inhibitor. Clin. Pharm..

[16] Sokolov V., Yakovleva T., Chu L., Tang W., Greasley P.J., Johansson S., Peskov K., Helmlinger G., Boulton D.W., Penland R.C. (2020). Differentiating the Sodium-Glucose Cotransporter 1 Inhibition Capacity of Canagliflozin vs. Dapagliflozin and Empagliflozin Using Quantitative Systems Pharmacology Modeling. CPT Pharmacomet. Syst. Pharm..

[17] Zeuthen T., Gorraitz E., Her K., Wright E.M., Loo D.D.F. (2016). Structural and functional significance of water permeation through cotransporters. Proc. Natl. Acad. Sci. USA.

[18] Evenepoel P., Bammens B., Verbeke K., Vanrenterghem Y. (2006). Acarbose treatment lowers generation and serum concentrations of the protein-bound solute p-cresol: A pilot study. Kidney Int..

[19] Evenepoel P., Meijers B.K., Bammens B.R., Verbeke K. (2009). Uremic toxins originating from colonic microbial metabolism. Kidney Int..

[20] Kanai Y., Lee W.S., You G., Brown D.A., Hediger M. (1994). The human kidney low affinity Na+/glucose cotransporter SGLT2. Delineation of the major renal reabsorptive mechanism for D-glucose. J. Clin. Investig..

[21] Mahaffey K.W., Jardine M.J., Bompoint S., Cannon C.P., Neal B., Heerspink H.J., Charytan D.M., Edwards R., Agarwal R., Bakris G. (2019). Canagliflozin and Cardiovascular and Renal Outcomes in Type 2 Diabetes Mellitus and Chronic Kidney Disease in Primary and Secondary Cardiovascular Prevention Groups. Circulation.

[22] Neuen B.L., Ohkuma T., Neal B., Matthews D.R., de Zeeuw D., Mahaffey K.W., Fulcher G., Blais J., Li M.Q., Jardine M.J. (2021). Relative and Absolute Risk Reductions in Cardiovascular and Kidney Outcomes with Canagliflozin Across KDIGO Risk Categories: Findings from the CANVAS Program. Am. J. Kidney Dis..

[23] McGuire D.K., Shih W.J., Cosentino F., Charbonne B., Cherney D.Z.I., Dagogo-Jack S., Pratley R., Greenberg M., Wang S., Huyck S. (2021). Association of SGLT2 Inhibitors with Cardiovascular and Kidney Outcomes in Patients with Type 2 Diabetes: A Meta-analysis. JAMA Cardiol..

[24] Heerspink H.J.L., Stefánsson B.V., Correa-Rotter R., Chertow G.M., Greene T., Hou F.-F., Mann J.F.E., McMurray J.J.V., Lindberg M., Rossing P. (2020). Dapagliflozin in Patients with Chronic Kidney Disease. N. Engl. J. Med..

[25] Bhatt D.L., Szarek M., Pitt B., Cannon C.P., Leiter L.A., McGuire D.K., Lewis J.B., Riddle M.C., Inzucchi S.E., Kosiborod M.N. (2021). Sotagliflozin in Patients with Diabetes and Chronic Kidney Disease. N. Engl. J. Med..

[26] Muskiet M.H.A., Heerspink H.J.L., van Raalte D.H. (2020). SGLT2 inhibitors: Expanding their Empire beyond diabetes. Lancet Diabetes Endocrinol..

[27] Agus A., Clément K., Sokol H. (2020). Gut microbiota-derived metabolites as central regulators in metabolic disorders. Gut.

[28] de Filippis F., Pellegrini N., Vannini L., Jeffery I.B., la Storia A., Laghi L., Serrazanetti D.I., di Cagno R., Ferrocino I., Lazzi C. (2016). High-level adherence to a Mediterranean diet beneficially impacts the gut microbiota and associated metabolome. Gut.

[29] Garcia-Mantrana I., Selma-Royo M., Alcantara C., Collado M.C. (2018). Shifts on Gut Microbiota Associated to Mediterranean Diet Adherence and Specific Dietary Intakes on General Adult Population. Front. Microbiol..

[30] Li Y.J., Chen X., Kwan T.K., Loh Y.W., Singer J., Liu Y., Ma J., Tan J., Macia L., Mackay C.R. (2020). Dietary Fiber Protects against Diabetic Nephropathy through Short-Chain Fatty Acid–Mediated Activation of G Protein–Coupled Receptors GPR43 and GPR109A. J. Am. Soc. Nephrol..

[31] Niewczas M.A., Sirich T.L., Mathew A.V., Skupien J., Mohney R.P., Warram J.H., Smiles A., Huang X., Walker W., Byun J. (2014). Uremic solutes and risk of end-stage renal disease in type 2 diabetes: Metabolomic study. Kidney Int..

[32] Poesen R., Claes K., Evenepoel P., de Loor H., Augustijns P., Kuypers D.R., Meijers B. (2016). Microbiota-Derived Phenylacetylglutamine Associates with Overall Mortality and Cardiovascular Disease in Patients with CKD. J. Am. Soc. Nephrol..

[33] Vanholder R., Schepers E., Pletinck A., Nagler E.V., Glorieux G. (2014). The Uremic Toxicity of Indoxyl Sulfate and p-Cresyl Sulfate: A Systematic Review. J. Am. Soc. Nephrol..

[34] Kikuchi K., Saigusa D., Kanemitsu Y., Matsumoto Y., Thanai P., Suzuki N., Mise K., Yamaguchi H., Nakamura T., Asaji K. (2019). Gut microbiome-derived phenyl sulfate contributes to albuminuria in diabetic kidney disease. Nat. Commun..

[35] Nemet I., Saha P.P., Gupta N., Zhu W., Romano K.A., Skye S.M., Cajka T., Mohan M.L., Li L., Wu Y. (2020). A Cardiovascular Disease-Linked Gut Microbial Metabolite Acts via Adrenergic Receptors. Cell.

[36] Ravid J.D., Kamel M.H., Chitalia V.C. (2021). Uraemic solutes as therapeutic targets in CKD-associated cardiovascular disease. Nat. Rev. Nephrol..

[37] Vince A.J., McNeil N.I., Wager J.D., Wrong O.M. (1990). The effect of lactulose, pectin, arabinogalactan and cellulose on the production of organic acids and metabolism of ammonia by intestinal bacteria in a faecal incubation system. Br. J. Nutr..

[38] Mishima E., Fukuda S., Kanemitsu Y., Saigusa D., Mukawa C., Asaji K., Matsumoto Y., Tsukamoto H., Tachikawa T., Tsukimi T. (2018). Canagliflozin reduces plasma uremic toxins and alters the intestinal microbiota composition in a chronic kidney disease mouse model. Am. J. Physiol. Physiol..

[39] Ho H.-J., Kikuchi K., Oikawa D., Watanabe S., Kanemitsu Y., Saigusa D., Ikeda-Ohtsubo W., Ichijo M., Akiyama Y., Aoki Y. (2021). Supplementary Data set v2.pdf for a paper entitled "SGLT-1-specific inhibition ameliorates renal failure and alters the gut microbial community in mice with adenine-induced renal failure. Physiol. Rep..

[40] Sirich T.L., Plummer N.S., Gardner C.D., Hostetter T.H., Meyer T.W. (2014). Effect of Increasing Dietary Fiber on Plasma Levels of Colon-Derived Solutes in Hemodialysis Patients. Clin. J. Am. Soc. Nephrol..

[41] Li L., Xiong Q., Zhao J., Lin X., He S., Wu N., Yao Y., Liang W., Zuo X., Ying C. (2020). Inulin-type fructan intervention restricts the increase in gut microbiome–generated indole in patients with peritoneal dialysis: A randomized crossover study. Am. J. Clin. Nutr..

[42] Meijers B.K., De Preter V., Verbeke K., Vanrenterghem Y., Evenepoel P. (2010). p-Cresyl sulfate serum concentrations in haemodialysis patients are reduced by the prebiotic oligofructose-enriched inulin. Nephrol. Dial. Transpl..

[43] Masereeuw R., Mutsaers H.A., Toyohara T., Abe T. (2014). The Kidney and Uremic Toxin Removal: Glomerulus or Tubule?. Semin. Nephrol..

[44] Breljak D., Ljubojević M., Hagos Y., Micek V., Eror D.B., Madunić I.V., Brzica H., Karaica D., Radović N., Kraus O. (2016). Distribution of organic anion transporters NaDC3 and OAT1-3 along the human nephron. Am. J. Physiol. Physiol..

[45] Chino Y., Samukawa Y., Sakai S., Nakai Y., Yamaguchi J., Nakanishi T., Tamai I. (2014). SGLT2 inhibitor lowers serum uric acid through alteration of uric acid transport activity in renal tubule by increased glycosuria. Biopharm. Drug Dispos..

[46] Andrade S.J., Flores Fonseca M.M. (2018). Renal Handling of Uric Acid. Contrib. Nephrol..

[47] Mihaila S.M., Faria J., Stefens M.F.J., Stamatialis D., Verhaar M.C., Gerritsen K.G.F., Masereeuw R. (2020). Drugs Commonly Applied to Kidney Patients May Compromise Renal Tubular Uremic Toxins Excretion. Toxins.

[48] Juni R.P., Al-Shama R., Kuster D.W., van der Velden J., Hamer H.M., Vervloet M.G., Eringa E.C., Koolwijk P., van Hinsbergh V.W. (2020). Empagliflozin restores chronic kidney disease–induced impairment of endothelial regulation of cardiomyocyte relaxation and contraction. Kidney Int..

